# Multilevel Optical Storage, Dynamic Light Modulation, and Polarization Control in Filamented Memristor System

**DOI:** 10.1002/adma.202411186

**Published:** 2024-11-20

**Authors:** Alexander Korneluk, Tomasz Stefaniuk

**Affiliations:** ^1^ Faculty of Physics University of Warsaw Pasteura 5 Warsaw 02–093 Poland

**Keywords:** electrochemical metallization, nonvolatile memories, optical memristors, optical modulators, polarization control

## Abstract

The electrochemical metallization (ECM) mechanism is emerging as a promising approach for the development of optical memristors—nonvolatile memory systems proposed for use as artificial synapses in neuromorphic computing applications. ECM memristors offer exceptional operating dynamics and power efficiency compared to other systems, but challenges with reproducible cycle‐to‐cycle state switching and the absence of advanced optical functionalities hinder their integration into photonic systems. In this work, an ECM free‐standing memristor structure is proposed, which simultaneously offers wavelength‐dependent multilevel nonvolatile optical storage, volatile light modulation, and dynamic polarization control. It is demonstrated that in the presence of a resonance, the optical readout provides noise‐free, robust, and significantly more accurate information about the memristor's state than electrical measurement. The use of light allows to gain insight into the intermediate electrical levels of the device as it transitions between high and low resistance states and to recover the complete record of applied voltages even when stochastic filament ruptures occur. Finally, the investigations show that spectroscopic ellipsometry provides real‐time information on the dynamics of cation movement and the corresponding permittivity changes at the interfaces between the switching layer and the electrodes, thus becoming a complementary characterization method for ECM memristors alongside state‐of‐the‐art techniques.

## Graphical abstract

The study proposes a multifunction metallic filamented memristor offering wavelength‐dependent optical storage, light modulation, and polarization control. The optical readout of the structure's state provides more accurate information than electrical measurements, enabling the recovery of the complete record of applied voltages even in the presence of stochastic filament breaks. Spectroscopic ellipsometry gives real‐time insights into cation dynamics and permittivity changes.

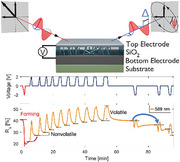

## 1 Introduction

The ability to vary the resistance in a nonvolatile manner gave rise to the concept of memristors—physically reconfigurable systems that can provide a paradigm shift in building highly efficient computing machines beyond classical von Neumann computing architectures.^[^ ^1^ ^]^ Such systems are particularly desirable in the world of neural networks, reservoirs, and neuromorphic computing, where fast data processing and efficient storage capabilities are critical.^[^ ^2^, ^3^, ^4^, ^5^ ^]^ One of the key features of memristive systems lies in their capacity to hold their resistive state without the need for an external power supply, thus greatly reducing the device's power consumption. Memristors can mimic synaptic responses, akin to the human brain, allowing the circumventing of the bottleneck of data transfer between computing unit and memory—a persistent challenge in traditional computing architectures.^[^ ^6^, ^7^ ^]^ Through this emulation, memristors have demonstrated superior performance and have been proven to simplify the memory hierarchy dramatically and enable compute‐in‐memory operations.^[^ ^8^, ^9^, ^10^ ^]^

While much attention has been focused on the properties and applications of conventional electrical memristors, research into their optical equivalents is gaining momentum. The optical memristors promise improved switching speed, higher power efficiency, and possible integration with existing optical communication technologies.^[^ ^11^, ^12^ ^]^ They also should offer advantages such as reduced Joule heat generation, minimal crosstalk, and parallel (multiwavelength) processing capability to overcome the limitations associated with purely electrical solutions.^[^ ^12^, ^13^ ^]^ However, in order to realize this potential, optical memristors must exhibit several key features. In their case, nonvolatile storage means that they should be able to maintain various states of transmission or reflection over a period of time without the need for an external power supply. The capability to operate across a continuous range of optical levels (analog operation) rather than binary would enable them to act as memristive synapses, thus facilitating multilevel memory and processing capabilities. Multilevel functionality is intrinsically linked to the desired deterministic storage and switching behavior, ensuring precise and consistent control over state changes in in‐memory operations. Endurance is another critical feature, allowing the memristor to withstand numerous read/write cycles under varying thermal conditions over its lifetime without performance degradation. The underlying physical switching mechanism should be energy efficient and fast to facilitate rapid data access and enhance overall system responsiveness. Finally, the system should be scalable and CMOS compatible, allowing memristors to be efficiently integrated into nanoscale devices and circuits.

Despite years of intensive research, designing and fabricating an optical memristor that meets all the desired criteria has proven to be a significant challenge.^[^ ^11^, ^12^ ^]^ In this pursuit, a variety of proposals have been put forth for resistive systems that can be read, written, or have their switching characteristics altered through the application of light.^[^ ^1^, ^14^, ^15^ ^]^ The advantages and disadvantages of certain types of reported optical memristors are, first and foremost, stem from the specific device‐switching mechanisms employed.^[^ ^1^, ^11^, ^16^, ^17^ ^]^ Given the described characteristics of an ideal optical memristor, the electrochemical metallization (ECM) mechanism emerges as one of the most promising approaches. The process involves the formation and dissolution of metallic filaments (conductive bridges, CBs) within an insulating layer, facilitated by the application of voltage.^[^ ^16^, ^18^ ^]^ This allows the device to switch between high‐resistance (HRS) and low‐resistance (LRS) states reversibly. ECM devices bear similarity to valence change memory memristors, as both rely on the formation of conductive CBs. However, ECM operation is governed by the migration of metal cations rather than oxygen vacancies, resulting in improved switching dynamics, a higher extinction ratio, and reduced current leakage.^[^ ^3^ ^]^ Compared to other systems in general, ECM‐based memristors are known for their very low current operation, efficient recovery of excellent insulator properties at HRS, and high on/off resistance ratio. They are also highly scalable and CMOS‐compatible.^[^ ^1^ ^]^

Leveraging the accumulated know‐how from resistive filamentation in conventional ECM memristors, variants of these devices that also support optical reading and writing capabilities have started to be developed. However, a measurable optical readout is a nontrivial task due to the reduced interaction area between an optical beam and the filaments. This is one of the reasons why most, if not all, of ECM optical memristors have been realized in waveguide geometry. The first demonstrations of optical readout of the resistance state were shown in ECM memristors integrated with plasmonic waveguides, which relied on the detection of absorption and scattering changes due to the presence or absence of metal filaments.^[^ ^19^, ^20^, ^21^ ^]^ Over time, the concept evolved into atomic‐scale plasmonic switches, where the optical properties are altered by the electrical manipulation of the individual or, at most, a few atoms. It has been shown that light can be used not only to read the state of the device^[^ ^22^ ^]^ but also to write to it.^[^ ^23^, ^24^ ^]^ Unfortunately, while ECM‐based memristors have often exhibited a number of electrical states, their optical functionality has so far been limited to just two levels.

The long‐range mobility of cations, a key feature of ECM memristors, contributes to their exceptional operating dynamics. However, due to the stochastic nature of filament growth, the nature of the mechanism also leads to their most significant drawback: a lack of reproducible device switching between cycle‐to‐cycle and device‐to‐device. High mobility causes node weight variation in memristor systems and reduces long‐term reliability, particularly at low conductance states and in long filaments because of weakly formed channels.^[^ ^25^, ^26^ ^]^ The interactions and formation of metal clusters are significantly influenced by kinetic factors, resulting in various shapes and growth modes of the clusters that can be observed experimentally.^[^ ^27^, ^28^ ^]^ Even though fine analog states can be modulated from these devices, they cannot be reliably preserved for computing applications. Different strategies have been proposed to tackle low voltage reliability issues in ECM. Some of the techniques, like initial electroforming^[^ ^29^ ^]^ or filament alloying,^[^ ^7^ ^]^ tried to solve the problem from the physical point of view by eliminating incomplete conduction channels or making the filament growth more stable. Others, like noise‐aware training^[^ ^30^ ^]^ or programming write‐verify schemes,^[^ ^31^ ^]^ aim to reinforce the system so that its performance remains largely unaffected even in the presence of device variations.

Despite the considerable effort that has been undertaken during the last few years to optimize existing memristor technologies, there is still a strong need for a high‐quality optical memristor system that offers excellent analog tunability while maintaining stability at every level of conductance. The situation is further complicated by the fact that even the description of the formation of CBs and the underlying physical and chemical mechanisms is still a matter of debate.^[^ ^32^ ^]^ The techniques typically used to investigate the filament growth process, such as transmission electron microscopy (TEM),^[^ ^27^, ^28^ ^]^ electron energy loss,^[^ ^33^ ^]^ conductive atomic force microscopy^[^ ^34^ ^]^ or X‐ray‐based spectroscopy, and crystallography^[^ ^35^ ^]^ usually compare only the before and after states and do not provide any information on the intermediate conditions of the system. Moreover, they require a vacuum environment that can also impact the behavior of the memristor,^[^ ^36^ ^]^ cover a small sample area, and give vague results stemming from poor contrast of imaging detectors.^[^ ^37^ ^]^

Our study proposes an alternative approach to achieve a highly reliable ECM‐based memristive system. We demonstrate that in structures with well‐defined resonance, optical readout provides significantly more accurate information about the resistance state of the memristor than electrical measurement. We show that optical states can retain the complete history of applied voltages, even in the presence of stochastic dendrite ruptures. This observation implies that the optical memristor can successfully operate in a low current density regime, which is typically unreliable in ECM‐based devices. Furthermore, our findings indicate that optical detection provides information on the intermediate states of the device as it transitions between the ON and OFF states. We exploit this property to create the first ECM optical memristor, offering nonvolatile and multilevel operation with reversible state changes, the capability for volatile light modulation, polarization ellipse rotation and modification, and wavelength‐dependent response—features that have not been reported together in a single memristor device of any type before. Furthermore, our investigations confirm that vacuum‐free spectroscopic ellipsometry can provide real‐time information on the dynamics of cation movement both in the switching layer and at the electrode interfaces. We have optically traced the transition from unstable to sustained CBs, and on the basis of a developed ellipsometric model, we reveal why the material symmetric memristor cell is capable of bipolar operation.

## 2 Results

### 2.1 Device Architecture and Operational Principle

The typical ECM memristor cell, reported in the literature, consists of an electrochemically active electrode such as Ag, Cu, or Ni, a switching layer, typically an isolator like SiO_2_, Al_2_O_3,_ or amorphous Si, and an electrochemically inert counter electrode such as Pt, Au, and W.^[^ ^16^, ^18^, ^27^, ^36^ ^]^ When the voltage is applied, atoms from the active electrode undergo ionization, migrate through an insulating layer, and are reduced on the inert electrode, forming a conductive bridge. The completion of a metal filament creates an electrically conductive path within the dielectric layer, resulting in an LRS of the memristor.^[^ ^16^, ^18^, ^27^, ^36^ ^]^ The reverse process, in which a filament segment is physically (Joule heating) and/or chemically (oxidative dissolution) disrupted, removing some of the filament material, initiates the reset process that transitions the device back to the HRS. As the cations do not chemically react with the host material in the switching layer, they can be completely removed from it when a reverse bias is applied.^[^ ^38^ ^]^ The thickness of the switching layer is also an important parameter, as thinner devices have been reported to suffer significantly less from resistive heating effects and can withstand higher currents before breakdown.^[^ ^26^ ^]^

**Figure 1:**
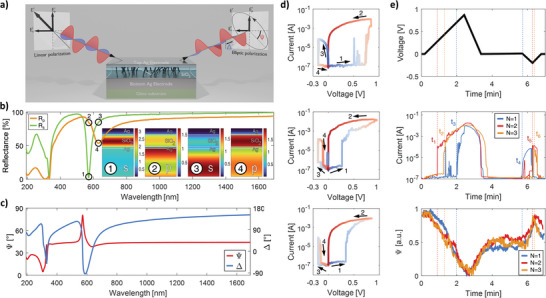
The general properties of the structure are under investigation. a) A schematic showing the device geometry and illustrating the principle of optical measurement. b) Reflectance spectra of a freshly prepared sample without filaments, measured at an illumination angle of 65° for p and s polarizations. The insets show the simulated electric field distributions across the structure for the wavelengths marked in the reflectance spectra (FDTD simulations). c) The ellipsometric parameters Ψ and Δ, measured at 65° for the same sample as in b). d) Current–voltage curves recorded for the first three cycles. Color transitions from blue to red signify the progression of time, and arrows show the direction of bias change. e) Time‐dependent voltage and current curves along with the corresponding Ψ curves.

Bearing these relationships in mind but aiming to optically investigate the structure under conditions where the ECM device is generally considered to operate unreliably, we designed our structure differently. The memory cell geometry, working principle, and optical setup configuration are schematically shown in **Figure** **1**a. The device consists of two active silver electrodes separated by an insulating layer of SiO_2_. The top and bottom electrodes are 20–22 and 100 nm thick, respectively, while the switching layer thickness is 186–189 nm (cell‐to‐cell variation of a few nm). The choice of Ag as the electrode material is due to silver's well‐known excellent optical properties, particularly its high reflectivity and low optical losses in the visible and NIR spectral regions.^[^ ^39^ ^]^ It was, therefore, expected that such a thick switching layer would create a high‐quality optical cavity, increasing the amount of information gathered from the active region. On the other hand, we knew from the literature that the high Ag cation mobility and thermodynamic instability in SiO_2_ in most devices resulted in significant resistance‐switching ratio variation and poor data retention, especially for long filaments.^[^ ^7^ ^]^ Such a choice of materials will thus be beneficial to the intended reliability analysis. The optical response of the sample was measured from the top electrode side, taking advantage of the semi‐transparency of the Ag layer. The area of the structure where the filaments were expected to grow was exceptionally large compared to other reported ECM devices, measuring 2.5 mm × 3.5 mm. On the one hand, such a large active area significantly improves the sensitivity of both electrical and optical readout, allowing for more precise measurements and the detection of subtle physical phenomena. However, the trade‐off is a reduction in operating speed. The larger active surface increases the overall capacitance of the device, leading to slower charge and discharge times. Nevertheless, this is not a significant issue, as ellipsometric measurements take approximately 1 second to acquire.

The assumptions regarding the optical properties of the structure were positively validated by polarization‐dependent intensity reflectance data and spectroscopic ellipsometry data (Psi (Ψ) and Delta (Δ) curves), both measured at 65° of incidence (Figure 1b). ψ signifies the amplitude ratio change between the p‐ and s‐polarized light components upon reflection, while Δ represents the phase difference between these components, together providing a comprehensive characterization of the change in light polarization. The freshly fabricated structure (with no filaments) exhibits a clearly visible optical resonance ≈580 nm for s polarization and 620 nm for p polarization, with a higher Q factor in the former case. As indicated by the insets, near the resonance, the electric field is primarily localized within the switching layer, precisely where filament growth is expected. The presence of resonance is also manifested in the ellipsometric curves and leads to a rapid change in the state of polarization of the reflected beam. The maximum value of Ψ occurs at a wavelength of 571 nm, whereas the minimum value of Δ is observed at 588 nm.

We then performed the first series of electrical and ellipsometric measurements under an applied voltage to verify whether the symmetrical structure exhibits nonvolatile and reversible electrical or optical characteristics. The issue was not immediately apparent, as typical ECM memristors have chemically asymmetric cells to facilitate the removal of cations from the switching layer when a reverse voltage is applied. Therefore, they function as bipolar switches, meaning the setting process is specific to one polarity, while the controlled reset process occurs exclusively at the opposite polarity.^[^ ^18^, ^40^ ^]^ The set of current–voltage (*I*–*V*) curves obtained during the first three cycles of the cell, without any preliminary electroforming procedure, is shown in Figure 1d. When the positive voltage is applied at the first stage of the first cycle (top row), the device is in HRS with almost no current flowing through the system (arrow #1). At ≈0.5 V, sharp spikes start to appear, indicating that some of the growing dendrites have reached the opposite electrode. However, the filaments are not yet stable, and the flowing current causes them to collapse. Above 0.7 V, the current begins to rise in a more stable manner until it reaches approximately 8.9 mA, and the device switches to LRS. The system remains in the LRS during voltage reduction (arrow #2) and even when reverse bias is applied (arrow #3). The reset occurs at ≈−0.2 V, when the device switches back to the HRS, rapidly decreasing the flowing current. The recorded hysteresis of the current–voltage curve confirms that the symmetric ECM cell has the potential to function as a memristive system with bipolar switching behavior (for additional examples of other cells exhibiting memristor type of behavior in several subsequent cycles, see Figure S1, Supporting Information). In the following cycles (middle and bottom rows in Figure 1d), the *I–V* characteristics measured for positive voltages look similar, with the only difference being that the switching to HRS occurs at lower voltages, ≈0.25 V. This is expected due to the increased ease of dendrite formation after the initial electroforming in the first cycle.^[^ ^38^ ^]^ However, at negative voltages (arrow #3), the current follows the LRS branch instead of the HRS, switching to the HRS only at higher negative voltages and only for a short time (arrow #4). This means that in both of these cycles, information about the history of the applied voltage is lost due to unwanted and random breaks in filament integrity. These failures become even more apparent when examining the current and voltage characteristics in the time domain, as demonstrated in Figure 1e. Even though similar voltage ramps were used in all cycles (top row), the current curves rise at different times and have different shapes (middle row). The measured optical signal, shown in the bottom row of Figure 1e, is extremely contrasting in this respect. It is free of similar instabilities and faithfully reflects both the amplitude and polarization of the applied voltage. This quality difference is due to the fact that complete dissolution of the dendrites is not necessary to stop current flow. Near the device's switching point, removing even a small number of atoms from the filaments can create nanometer‐sized gaps sufficient to disrupt electrical contact between the electrodes, thereby significantly affecting the *I*–*V* characteristics. In contrast, such small, sub‐wavelength gaps have little effect on the optical signal, as the total amount of Ag ions in the SiO₂ layer remains largely unchanged.

### 2.2 Ellipsometric Investigations of the Switching Mechanism

**Figure 2:**
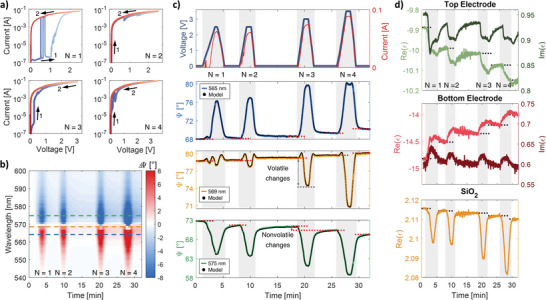
The device's electric and optical response to positive voltage. a) Current–voltage curves recorded for the first four cycles. b) ΔΨ changes as a function of time and wavelength. The dashed line marks the selected wavelengths representing different types of changes, which have been chosen for further analysis in the middle column graph set. c) The time dependence of current and voltage, along with the corresponding Ψ functions, measured for wavelengths of 565, 569, and 575 nm, respectively, and are shown together with the developed model (black dotted line). d) The permittivities of the top and bottom electrodes and the switching layer for a wavelength of 575 nm are all extracted from the ellipsometric model. The Ψ curves were collected under illumination at 65°. The shaded areas in (c) and (d) represent the time interval at which the voltage was applied.

To study the correlations between the recorded optical and applied electrical signals and to understand the underlying mechanism of bipolar switching, we divided further research into case studies with either only positive or negative voltage (the terms refer to the bias sign applied to the bottom electrode). In **Figure** **2**a, we present the measured I‐V curves for four subsequent cycles of a fresh cell. The device remains in LRS, except for the first stage of the first cycle (arrow #1), as we have applied only positive voltage to the bottom electrode. At first glance, the measured current values may seem large and capable of causing thermal effects in the structure. However, this impression can be misleading when considering the size of the active area. In our sample, the current density in LRS is ≈10^−8^ A µm^−2^ (≈10^−14^ A µm^−2^ in HRS), which is significantly lower than the typical ≈10^−4^ A µm^−2^ (≈10^−13^ A µm^−2^ in HRS) reported in the literature for similar ECM systems.^[^ ^41^ ^]^ Thus, while the influence of thermal effects on the formation and dissolution process of the filaments cannot be ignored, they should not be more pronounced than in other structures (see also discussion in Section S1.3, Supporting Information).

In Figure 2b, we present the associated time‐dependent optical signals, expressed as changes in the ellipsometric parameter Ψ, thereby providing information on the orientation of the polarization ellipse. Depending on the wavelength, the observed ΔΨ fluctuations are either positive (red) or negative (blue), with an ambiguous region in between. The strongest changes occur near the optical resonance (see Figure 1c) and diminish for wavelengths farther away from it. The variations in the Ψ parameter are related to the spectral shift of the resonance and change in its intensity, both effects induced by the applied voltage (see Figure S2a, Supporting Information). The sign of ΔΨ depends mainly on whether the spectral distance between the particular wavelength and the resonance center has decreased or increased as a result of the resonance shift.

In Figure 2c, we present detailed graphs of the current and voltage time courses, along with the corresponding Ψ functions for selected wavelengths, representing all three types of changes. In the initial phase of the first electrical pulse, when the device is still in the HRS, the current curve exhibits minimal variation, with only a few current peaks discernible. Meanwhile, in the optical curves, particularly at wavelengths of 565 nm, slight changes are already being detected, and the signal does not return to entry level even when the current peaks disappear. Then, when the device switches to the LRS state, the current starts to increase, first quickly and then in proportion to the applied voltage. With the exception of the Ψ curves obtained for the resonant wavelength, the other optical curves follow this trend and closely reflect the current curve. As the device remains in the LRS during subsequent cycles, the current and Ψ curves correlate with the applied voltage from the start of each cycle. However, another detail deserves attention. At the end of each cycle, the Ψ curve settled at a level different from its starting point. In practice, this means that even though the device remained in the same electrical state, we were able to observe different optical substates, induced by the applied voltage. In other words, the optical readout allowed us to retrieve the history of the applied electrical pulses, while this information was lost in the current measurements.

The changes induced by the applied voltage are also reflected in the time courses of Δ curves (Figure S3a,c, Supporting Information). Due to the different characteristics of the extremum occurring in the Δ curve, which has a much greater spectral width than the resonance in the PSI curve and a different shape, the changes seen in the Δ values occur for a wider range of wavelengths. Due to the distinct characteristics of the extremum in the Δ curve, which exhibits a much broader spectral width and a different shape compared to the resonance in the Ψ curve, the changes in Δ values occur over a wider range of wavelengths. The phase change can be either positive or negative; however, unlike Ψ, negative phase changes are far more prevalent, with changes reaching up to 120°. From this point on, changes that are characterized by a large amplitude that only occurs when the voltage is applied will be referred to as volatile changes. Conversely, changes related to a value that is different before and after the voltage cycle is applied will be called nonvolatile changes.

Utilizing the capabilities of ellipsometry, we have developed a model that accurately reproduces the measured Ψ curves (e.g., dotted black lines in Figure 2c) and provides information on the electrical permittivity values (Figure 2d) and thicknesses of the individual layers (Figure S5, Supporting Information) for a fresh cell and during subsequent cycles. It is worth noting that even at the measurements' starting point, the permittivity values differ from those of the bulk material (Figure S4, Supporting Information). This is particularly evident in the near‐infrared spectral region for the SiO₂ layer, where the curvature of the permittivity curve shifts, resembling a Drude‐like profile. According to ref. [37] one possible explanation is that the Ag clusters may have migrated spontaneously from the bottom electrode into the SiO_2_ layer during or shortly after its e‐beam deposition. Silver atoms introduced through processes such as thermal diffusion, interfacial reactions, or knock‐on effects, as discussed at the end of this section, may affect the device's current levels and influence filament growth dynamics.^[^ ^27^ ^]^ The deposition of a top silver electrode did not produce a similar effect (Figure S4, Supporting Information).

The developed ellipsometric model indicates that applying a positive voltage to the bottom electrode does not significantly impact the effective thickness of the individual layers. However, it increases the bottom electrode's permittivity while decreasing that of the top electrode, which seems to confirm earlier TEM‐based reports of conducting filament growth.^[^ ^27^, ^28^ ^]^ The bottom electrode seems to become “less metallic” due to void formation triggered by the oxidation and migration of metal clusters. At the same time, Ag ions are reduced when they reach a cathode, filling potential voids or gaps in the structure of the top electrode and improving its electric and, thus, optical performance. The most intriguing changes are in the effective optical properties of the SiO_2_ layer. Each time a positive voltage is applied, the electrical permittivity alters by more than 1% at 575 nm wavelength. Importantly, in contrast to other promising effects, such as free‐carrier accumulation,^[^ ^42^ ^]^ which are spatially confined to a few nanometers near the interface, the ECM‐based modulation exhibits a more volumetric character. Last but not least, a unique feature observed is that some changes in permittivity across all considered layers are nonvolatile and persist even after the voltage is removed. This confirms that the previously mentioned optical storage of past applied voltages is preserved as different permittivity values within the materials involved.

**Figure 3:**
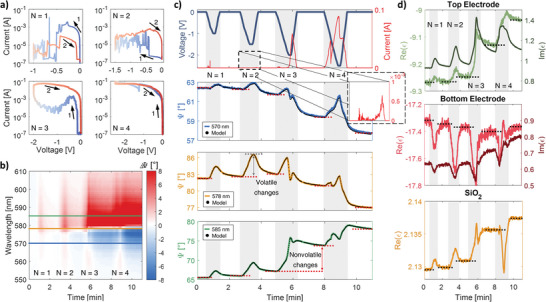
The device's electric and optical response to a negative voltage. a) Current–voltage curves recorded for the first four cycles. b) ΔΨ changes as a function of time and wavelength. c) The time dependence of current and voltage, along with the corresponding Ψ functions, measured for wavelengths of 570, 578, and 585 nm, respectively, and are shown together with the developed model (black dotted line). d) The permittivities of the top and bottom electrodes, as well as the switching layer, for a wavelength of 585 nm, are all extracted from the ellipsometric model. The Ψ curves were collected under illumination at 65°.

**Figure** **3** shows a comparable set of relationships obtained for negative voltages using a fresh cell. This time, to highlight the distinct characteristics of cation migration in this configuration, we gradually increased the applied voltage over successive cycles, which initially resulted in noisier current–voltage graphs (Figure 3a). Aside from some peaks in the first two cycles, the current level stayed close to the noise threshold, and the device remained in the HRS (see also the top graph in Figure 3c). The resistance switch occurred in cycle three, and after that, the device also remained in the LRS throughout cycle four. The use of such a procedure has allowed us to make some further observations. For negative voltage, the general impression is that the map showing fluctuations in Ψ values has been horizontally reversed, now with mostly positive changes occurring for longer wavelengths and negative ones for shorter wavelengths (Figure 3b). However, a closer analysis shows that, for the first two electrical pulses, irrespective of the wavelength, the application of voltage resulted in a permanent increase in Ψ function (Figure 3c). At the same time, the nonvolatile changes occur in two directions: a gradual decrease in the Ψ value for long wavelengths and an increase in the Ψ value for short wavelengths (see the supporting red dotted lines). This trend seems to break down when the cell starts to switch to the LRS in the third cycle, and current starts to flow through it. At this point, the direction of the volatile changes for short wavelengths reverses, while it remains consistent for nonvolatile changes. Analysis of the positions and amplitudes of the Ψ resonance peak indicates that its response to bias differs at negative voltages (Figure S2b, Supporting Information). Applying a voltage results in a much stronger increase in peak amplitude, even when the current flow is below the level of single milliamperes. This demonstrates the device's high sensitivity in this voltage configuration to the dendrite formation process. Additionally, a continuous shift in the resonance peak position toward longer wavelengths is observed. Similarly, the Δ changes observed for negative voltages (Figure S3a,c, Supporting Information) exhibit several distinct features when compared to those observed for positive voltages. As with the case of Ψ, the map of Δ changes for negative voltage is horizontally reversed, indicating that phase shifts are now positive for shorter wavelengths and negative for longer ones. Of particular note is the amplitude of Δ changes when the memristor starts to switch to LRS, which can reach up to +150°or down to −270°.

The characteristics of the Ψ and Δ functions described above are reflected in the permittivity curves shown in Figure 3d. Reversing the voltage polarity changed the direction of cation flow, leading to an increase in the electrical permittivity of the top electrode and a decrease in the bottom electrode in subsequent cycles. What is more notable is the shape of the SiO_2_ layer permittivity curve extracted from the ellipsometric model. There is a clear trend that nonvolatile changes always lead to an increase in the ɛ value, which is completely opposite to the situation with a positive voltage, while the sign of volatile changes alternates with each cycle. This is surprising as the structure of the cell is symmetrical, and we would therefore expect that there would be no differences depending on the polarity of the voltage. This nontrivial behavior can be explained within the framework of the qualitative electrochemical model proposed by Yang,^[^ ^27^ ^]^ which identifies different filament growth modes that can coexist within the same ECM system (see also extended discussion in Supporting Information). According to the theory, depending on ion mobility, redox rate, and ion supply, the forming filaments may vary in shape, formation direction, and dynamics. Our proposed memristor system is symmetric in terms of the materials used but asymmetric in geometry, specifically in the thickness of the electrodes. The 100 nm thick bottom electrode acts as a nearly infinite cation reservoir, enabling a high redox rate and causing a rapid influx of Ag ions into the SiO₂ layer under positive voltage, with elevated Ag concentration even after voltage removal (Figure 2d). In contrast, the 20 nm top electrode provides a limited ion supply due to its thin structure, surface roughness, and Ag's tendency to form islands^[^ ^43^ ^]^ (see also Figure S6, Supporting Information), impacting ion supply efficiency and thus filament growth mode. With negative voltage on the bottom electrode (Figure 3d), the system enters a bootstrapping growth mode, where voids left by migrating Ag clusters are inadequately refilled, increasing SiO₂ permittivity in the first two cycles. When electrode connections form, current flow modifies the growth mode, efficiently pumping Ag ions and reducing permittivity (cycles 3 and 4, volatile changes). However, when the voltage decreases, the system reverts to the previous growth mode, clearing Ag clusters from the SiO₂ and raising its permittivity again (cycles 3 and 4, nonvolatile changes).

It is also worth mentioning that the relationships described in the section above are general in nature. Wavelength‐dependent multilevel nonvolatile optical storage and volatile light modulation can also occur in “classical” ECM memristor systems with an inert electrode, provided the structure exhibits well‐defined optical resonance. To confirm this, we repeated the experiments for an Ag/SiO₂/Au cell and obtained qualitatively identical results (see Figure S7, Supporting Information). Both volatile and nonvolatile changes were detected, and notably, in this configuration under negative voltages, we also observed a reversal in the direction of volatile changes as the current increased (cycle 1 versus cycles 2 and 3).

### 2.3 The Development of Multilevel Optical Memristor

**Figure 4:**
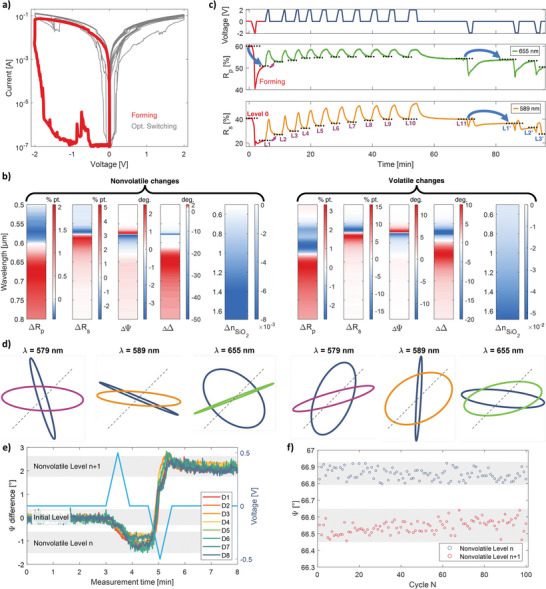
Performance of symmetrical optical memristor. a) Current–voltage curves recorded for the first 14 cycles. b) The train of electrical pulses (top row) together with the corresponding reflection coefficients measured for p (middle row) and s (bottom row). c) A synthesized summary of averaged nonvolatile and volatile changes in the optical properties in a single switching operation. The data is presented for two polarizations (p and s). d) Examples of changes in the orientation and shape of the polarization ellipses plotted for selected wavelengths and switching cycles (illumination at 65^O^). The three graphs on the left correspond to nonvolatile operation, while the three on the right correspond to volatile operation (different switching cycles were chosen for both cases). The gray line indicates the input polarization state of the illuminating beam, the blue lines show the output polarization state with no voltage applied, and the colored lines depict the output polarization state either with the applied voltage (volatile) or immediately after the voltage is removed (nonvolatile). Device‐to‐device (e) and cycle‐to‐cycle (f) variations in switching performance, expressed as changes in Ψ functions.

Finally, considering the relationships described above, we examined whether the observed nonvolatile changes in optical properties were reversible and if this mechanism could potentially be used to create an analog optical memristor. The fresh cell underwent a series of electrical pulses, with the resulting current–voltage curves from subsequent cycles presented in **Figure** **4**a. The pattern consisted of both positive and negative voltages, ensuring a comprehensive examination of the structure's response to different electrical stimuli. Judging from the shape of the *I–V* curves, the structure switched to LRS in the first cycle and remained in this state throughout the rest of the test procedure.

In Figure 4b, we present the detected changes in reflection under 65° illumination for two orthogonal polarizations, p, and s, at wavelengths where the absolute variations in value are the most pronounced. They are only a subset of a broader family of recorded curves with different characteristics (see also Figure S8 and the discussion in Section S1.2, Supporting Information regarding the influence of pulse parameters on the optical response of the memristor). A number of key insights can be derived from the data presented. First, the recorded global changes in reflectivity are remarkably large for a device with such a simple structure and relatively small variations in the permittivity values of the individual layers, accumulating even 30% for s‐polarized light and ≈17% for p‐polarized light. The difference in the results obtained for the two polarizations can be attributed to the depth of resonance in the reflection curve (see Figure 1b). Second, the direction of the nonvolatile (as well as volatile) changes depends directly on the sign of the applied voltage and can be reversed at any time. This feature allows the device to meet the condition of analog optical operation. Third, the strength and the characteristics of the changes and, thus, the device's optical response depend largely on the wavelength of the radiation used and its polarization. Fourth, one can also optically distinguish the moment of electrical switching between the LRS and HRS, from the amplitude of the optical changes (first cycle). Lastly, the optical curves exhibit significantly higher repeatability in subsequent cycles and offer lower noise levels compared to the current curves (Figure 4a) even though the optical signal is gathered from a 30 times smaller area than electrical (see methods).

## 3 Discussion

To provide a comprehensive overview of the optical properties of the developed system, Figure 4c presents a synthesized summary of its performance. As the previously given data anticipated, the most significant changes in the optical response occur near the resonance position specific to a given polarization, with volatile changes characterized by higher amplitude than nonvolatile ones. The extinction ratio calculated from reflection values in a single switch cycle for an optimum wavelength and s polarization is 3.5 dB µm^−1^ (1.75 dB µm^−1^ for nonvolatile), similar to ECM‐based waveguide systems.^[^ ^21^ ^]^ The rotation of the polarization ellipse from negative to positive voltage equals 25° (6° for nonvolatile) and the phase shift 30° (52° for nonvolatile). The extracted from an ellipsometric model change in the SiO_2_ refractive index value Δn increases with the wavelength and can be as large on average as 0.052 (0.008 for nonvolatile). We associate this dependence with the Drude‐like metallic response of metal clusters present in the volume of the switching layer. To put the derived Δn value in a broader context, it is worth introducing a figure of merit (FOM), which is equal to the ratio of the change in the index to the required power density. For the operation mode proposed above, when the device is in a low resistance state, and the current flows through the structure, the FOM is ≈0.019 cm^2^ W^−1^ (at 1550 nm wavelength). However, when the device is in off mode (see, e.g., cycle #2 in Figure 3, Δn ≈ 0.06 at ≈0.008 W cm^−2^ at 1550 nm wavelength), FOM can be as high as ≈7.5 cm^2^ W^−1^. The value is considerably higher than that observed in the Kerr effect^[^ ^44^ ^]^ FOM ≈ 10^−16^ cm^2^ W^−1^ (Δn ≈ 0.07 at 20 × 10^12^ W cm^−^^2^) and comparable to that obtained for the most energy‐efficient phase change materials, such as gallium^[^ ^45^ ^]^ FOM ≈ 3 cm^2^ W^−1^ (Δn ≈ 1.5 at 0.3 W cm^−2^).Yet, in contrast to the latter, the materials we have used are compatible with CMOS technology. While the absolute power efficiency record is held by systems based on carrier‐induced changes in refractive index ≈10^5^ cm^2^ W^−1^ (Δn ≈ 1.5 at 10^−5^ W cm^−^^2^), the proposed ECM‐based device has the potential to offer greater modulation capabilities given the same device geometry, as the index change effect occurs throughout the entire volume of the switching layer, not just in the proximity of the interface.^[^ ^42^ ^]^

Resonance‐enhanced optical readout also alters the energy required for a single switching event in the memristor. The measurement sensitivity allows the device to operate in a minimal‐current regime, even when the two electrodes are not electrically connected (see, for example, cycle #2 in Figure 3), by detecting subtle shifts in the position of silver nanoclusters within the switching layer. Under this approach, the energy efficiency of a single switching event, normalized to the surface area, reaches ≈0.1 nJ µm^−^^2^. This indicates that, despite the proposed structure not being specifically optimized for energy efficiency, the achieved values are comparable to or even lower than those reported for other nonvolatile electro‐optical or all‐optical memories based on ECM, PCM, ferroelectric, or MEMS effects.^[^ ^11^, ^46^ ^]^

To illustrate the influence of the complex relationship between Ψ and Δ changes on the shape and orientation of the polarization ellipse, we present examples in Figure 4d showing changes in the orientation and shape of the polarization ellipses plotted for selected wavelengths and switching cycles. These examples highlight how observed variations in Ψ and Δ can significantly impact the polarization state of light, either temporary (volatile change) or permanent (nonvolatile change), demonstrating the dynamic nature of polarization modulation under different conditions. To show the possible variety of options, we decided to illuminate the sample at 65° with an equal combination of p‐ and s‐polarization components (grey lines). Due to the presence of resonances, the structure intrinsically modifies the polarization of the incident beam, which results in different polarization states of the reflected beam (blue lines). The resonance selectively enhances, attenuates, or retards certain electromagnetic field components within the structure, leading to shifts in the polarization state. This effect depends on factors such as the angle of incidence, wavelength of the incoming light, and the intrinsic properties of the resonant structure, including cavity thickness and material refractive index.^[^ ^47^, ^48^ ^]^ In our case, the application of bias causes further changes. In some of the presented graphs, the applied voltage induces a polarization transition from a nearly linear to elliptic or even circular, and for others, vice versa (colored lines). The final result is significantly influenced not only by the chosen wavelength, but also, due to the different baseline values of Ψ and Δ in successive cycles, also by its sequence.

Lastly, since reliability is one of the well‐known challenges in ECM switching devices and is critical for their application in nonvolatile memory and neuromorphic computing, we have performed device‐to‐device, cycle‐to‐cycle, and long‐term stability tests of our devices. At this point, it is worth pointing out that, in terms of electrical performance, the proposed device represents a typical example of ECM‐based memristors, inheriting all limitations associated with the stochastic nature of filament growth. However, the optical readout, combined with the structure's strong optical resonance, effectively mitigates these drawbacks, offering enhanced stability and reliability. In Figure 4e, we present device‐to‐device variability expressed as changes in Ψ functions of eight different, freshly fabricated devices. It is noteworthy that, despite the absence of initial electroforming, typically employed in purely electrical memristors,^[^ ^38^ ^]^ and the use of relatively low‐voltage pulses (±0.5 V), the resulting curves exhibit remarkable consistency. All three nonvolatile optical levels are distinctly separated and clearly distinguished for all devices. Relative to the absolute Ψ value, fluctuations are ≈0.35% for level n and 0.2% for level n+1. This stability is not observed in the recorded current curves (Figure S9, Supporting Information). The current flow begins across devices at different times, and the corresponding resistivity values vary by ≈50%. The device's resilience to repeated switching cycles (+0.3 V and −0.5 V) for two closely positioned, nonvolatile optical levels is illustrated in Figure 4f. After 100 cycles, the optical states remained well‐separated, though device performance could likely be further optimized by fine‐tuning the positive and negative voltage amplitudes to better compensate for the differing characteristics of polarity‐dependent filament growth regimes. Finally, Figure S10 (Supporting Information) presents long‐term stability tests of nonvolatile optical levels switched on under either positive or negative voltages. After removing the electrical stimulus, the Ψ curves remained stable over a measurement period exceeding one hour, with distinct optical levels consistently well‐separated and unaltered.

## 4 Conclusion

In summary, our research confirms that ECM‐based devices exhibit significant potential as strong candidates in the quest for an ideal optical memristor system. In addition to the recognized advantages of filamented devices, such as exceptional operating dynamics and power efficiency, our experimental results demonstrate that utilizing optical resonance in these structures enables reproducible cycle‐to‐cycle state switching and noise‐free readout. Our findings indicate that optical methods not only surpass the precision of electrical measurements but also allow the retrieval of the complete record of applied voltages, even in the presence of stochastic filament ruptures. Furthermore, we demonstrate that choosing an ECM‐based system does not mean sacrificing advanced functionality. Our structure not only exhibits nonvolatile analog memory operation but also has unique features for an optical memristor system in general, including wavelength‐dependent response, the ability for volatile light modulation, and polarization modification capability. Through the use of resonance‐enhanced optical readout, we achieved reliable device‐to‐device and cycle‐to‐cycle performance. Finally, we prove that ellipsometry can provide real‐time information on the dynamics of cation movement, dendrite formation, and corresponding changes in permittivity values in the switching layer and electrode interfaces. By facilitating this technique, we were able to show that due to the different thicknesses and microstructure of the electrodes, the redox processes can vary between the top and bottom, which also affects the cation migration in the active layer. Consequently, we demonstrated that a symmetric filamented structure can exhibit bipolar switching, similar to asymmetric ones. We believe that our results are of a general nature and that they can be effectively applied to photonic systems with different geometries and with higher Q‐factors. The optical readout can be applied alongside other techniques that have already been proposed to solve the reliability issues in ECM‐based devices.

## 5 Experimental Section

### Sample Fabrication

The device was fabricated entirely using an electron beam PVD system (Lesker 75). The process began with the preparation of a commercial glass substrate (Ossila ultra‐flat quartz‐coated glass), which was cleaned using isopropanol, deionized water, and oxygen plasma to remove any residual dust particles. Subsequently, a 100 nm bottom silver electrode, a 200 nm SiO_2_ layer, and a 20 nm top silver electrode were deposited sequentially. These deposition processes were conducted under standard evaporation conditions at room temperature (22 °C) and a 5 × 10^−5^ Torr pressure. To arrange the layers, 3D‐printed shadow masks were employed, which were swapped after each layer to achieve the desired geometry. The mask‐changing process was performed in a laminar flow chamber to prevent any contaminants from settling on the surface of the layers. Each sample consisted of six pixels (areas where all layers overlap, ≈2.5 × 3.5 mm), with separate electrical contacts (pure indium) soldered to the bottom electrode of each pixel. The top electrode was common to all pixels, with only one electrical contact soldered to this layer.

### Ellipsometric Measurements and Modeling

The investigations on the optical properties of memristor were conducted with an RC2 ellipsometer (manufactured by J.A. Woollam Co.) equipped with dual‐rotating compensators placed before and after the sample. All data were collected with attached focusing probes, which reduced the beam diameter at the surface of the sample to ≈300 µm. A typical measurement procedure for each pixel was as follows. First, before applying any voltage, the Ψ and Δ values were measured for three angles of incidence: 60°, 65°, and 70°. This measurement was the starting point for creating a model of the structure and its interaction with light. Next, during the flow of electric pulses, real‐time measurements of ellipsometric parameters at a single angle of 65° for each voltage step, with 1s optical data acquisition time were performed. Finally, at the end of the series, the measurement at three angles were repeated to verify that the model based on the single‐angle measurement remained consistent. An intensity reflectance measurement accompanied each Ψ and Δ measurement throughout the procedure.

Then the construction of the layered model of the sample structure and dielectric permittivity function was proceeded. From the initial test, it turned out that the basic model of effectively homogeneous three layers (top electrode, switching layer, bottom layer) with some roughness on the top was sufficient to capture all the nuances of the material's behavior. More advanced models, e.g., with graded films, mixed materials, or additional layers, did not significantly improve the quality of the model in terms of MSE (mean squared error) value. To obtain the dielectric permittivity function, the general oscillator approach for the electrodes (three harmonic oscillators and Drude term), and the three‐term Sellmeier formula for the SiO_2_ layer was employed. The quality of the proposed model, and therefore of the analysis presented, was validated based on the MSE values, which were ≈6.5 for three‐angle measurements and ≈2.5 for single‐angle measurements.

### Electrical Characterization

For the *I–V* measurements, a voltage was applied to the selected pixel using a Rohde and Schwarz HMP 2020 power supply with a precision of 0.001 V. The current was measured with a Keithley Digital Multimeter 2701, and the compliance current was set to 200 mA. Each data point was acquired over approximately 0.2 seconds. In the initial series of measurements (Figure 1d,e), the voltage signal had a triangular shape with limits just above the resistance switch. During the second (Figure 2), third (Figure 3), and fourth (Figure 4) series of measurements, the voltage was gradually increased to observe changes in the ellipsometric curves. The voltage was held at a constant value when the Ψ curve started to saturate. Once the voltage cycle concluded and the Ψ value had stabilized, subsequent voltage pulses were initiated.

### FDTD Simulations

Finite difference time domain simulations were performed using Ansys Lumerical FDTD software. A 2D geometry was used, and the model was provided with optical constants and geometric parameters obtained from the ellipsometric measurements.

## Conflict of Interest

The authors declare no conflict of interest.

## Supporting information



Supporting Information

## Data Availability

Data openly available in public repository with DOI ans in Manuscript SI.
