# Supplementary material for: Multilevel Optical Storage, Dynamic Light Modulation, and Polarization Control in Filamented Memristor System
Source: Adv Mater. 2024 Nov 20;37(3):2411186. doi: 10.1002/adma.202411186 (PMC11756036; doi:10.1002/adma.202411186)
Supplement: Supplementary file 1 — Supporting Information [file ADMA-37-2411186-s001.docx]

Supporting Information

Multilevel optical storage, dynamic light modulation, and polarization control in filamented memristor system

Alexander Korneluk, Tomasz Stefaniuk*

E-mail: [tomasz.stefaniuk@fuw.edu.pl](mailto:tomasz.stefaniuk@fuw.edu.pl)

1.1. Electrochemical model of cluster migration

The observed ion migration can be explained within the qualitative electrochemical model framework proposed in the study [27], which reconciles different and seemingly contradictory experimental results. The model is based on the observation that metal clusters in the volume of a dielectric matrix behave as bipolar electrodes (BPEs) and can dissolve from their original locations and nucleate and redeposit at new positions. Depending on the kinetic factors of electrochemical redox reactions and ionic drift, the authors identified different filament growth modes in which filaments vary in shape, formation direction, and dynamics. In regimes characterized by high ion mobility (μ) and redox rate (Γ), ions reach the counter electrode without agglomerating, thereby preventing nucleation within the insulating film. This leads to filament growth initiating from the cathode, with the ample ion supply from the anode forming an inverted cone-shaped filament. In contrast, ions accumulate in environments with low ion mobility and redox rates, achieving the critical nucleation conditions within the dielectric. This so-called bootstrapping filament growth mode is dynamic and involves repeated cycles of cluster nucleation, merging, and migration toward the cathode electrode. As nanoclusters grow, they may leave behind voids that are subsequently filled by newly formed clusters, resulting in a continuous, progressive filament formation from the anode side. In general, μ determines the nucleation sites and the direction of the ﬁlament growth, while the Γ determines the ion supply and the geometry of the ﬁlament. The local Γ and μ can be affected by variations in the local temperature, defect density, defect energy proﬁle, microstructure, electrochemical potentials, electric ﬁelds, charge compensation, and the amount of available atoms/ions.^[27]^ It is also worth noting that different growth models may occur simultaneously within the same system, or a sudden transition between them may occur when the flowing current causes a rise in temperature, which significantly increases both μ and Γ. This is particularly true for switching layers made of SiO₂, an amorphous insulator, where the conditions for ion migration and nucleation are inhomogeneous and may vary throughout its volume.

Our proposed memristor system is symmetric in terms of the materials used but asymmetric in geometry, specifically in the thickness of the electrodes. The bottom electrode, with a thickness of 100 nm, can be considered a virtually infinite Ag reservoir relative to the volume required for filament formation. In this situation, with a high redox rate (Γ), applying a positive voltage causes a rapid influx of a large number of Ag ions into the SiO₂ layer, and the elevated concentration of Ag persists even after the voltage is turned off (Figure 2d). In contrast, the top electrode is only 20 nm thick and is deposited on a two-layer stack, with its surface roughness increased by the texture of the underlying films. The homogeneity of the top electrode is also affected by the fact that silver tends to agglomerate into islands,^[43]^ (see Figure S6). These factors may influence the efficiency of ion supply and, consequently, the preferred growth mode. It is also possible that the consumption of top electrode material may no longer be negligible. The described relationships become evident when a negative voltage is applied to the bottom electrode (Figure 3d). The system operates in bootstrapping growth mode, but due to insufficient ion supply, the voids that are left behind as metal clusters migrate toward the cathode, are no longer efficiently refilled. The outflow of silver clusters from the SiO₂ matrix causes an increase in the permittivity of the SiO₂ layer, as observed in the first two cycles, for both volatile and nonvolatile changes. The situation shifts when a connection is established between the electrodes, and the resulting current flow alters the growth mode, leading to efficient pumping of Ag ions into the SiO₂ layer and a decrease in permittivity (cycles 3 and 4, volatile changes). However, when the voltage decreases, the system reverts to the previous growth mode, eventually causing the permittivity of SiO₂ to increase again (cycles 3 and 4, nonvolatile changes). We believe that the differences between the two filament growth models are responsible for the observed reset process.

**1.2. Influence of pulse parameters on memristor optical response**

The physical operating mechanisms of the proposed Ag/SiO_2_/Ag memristor are consistent with those reported in the literature for traditional, purely electrical ECM-type memristors, as in both cases, ion migration occurs within the active layer. For this reason, there shouldn't be any significant differences, e.g., in terms of endurance or switching speed. The key distinction in our approach lies in the application of an alternative detection method—optical readout enhanced by resonance. And, in fact, since optical readout provides a more accurate insight into the state of the memristor, its characteristics may differ in certain aspects from those of electrical readout methods.

In **Figure S11a**, we present the impact of electrical pulse duration on the volatile and nonvolatile changes in the Ψ function. The memristor is stimulated by pairs of pulses with positive (+0.3 V) and negative (-0.5 V) voltages, with the amplitude carefully selected to ensure that the Ψ values at the end of each cycle do not deviate significantly from the initial state. This approach was intended to avoid saturation effects, which will be discussed later in the analysis. The pulse durations for each pair were set to 50 s, 20 s, 10 s, 5 s, and 2 s, respectively. The minimum pulse duration was constrained by the minimum time required for the ellipsometer to measure the Ψ function. The presented dependencies indicate that shorter pulse durations result in smaller volatile and nonvolatile changes. This relationship is unsurprising, as shorter pulses restrict the time available for ion migration and accumulation, processes essential for structural modifications. This factor should be carefully considered in the design of devices intended for ultra-fast switching, as it may be compensated, for example, by using pulses of higher amplitude.

**Figure S11b** provides a synthesized view of the influence of pulse amplitude on the magnitude of changes in the Ψ function. Several points are noteworthy. The nonvolatile changes for negative polarity are consistently larger than those for positive polarity, confirming distinct dendritic growth mechanisms for each polarity. This observation suggests that a more efficient approach to achieving permanent changes in silver nanocluster concentration within the SiO₂ layer is through bootstrapping-type growth in an insufficient ion supply regime.^[27]^ However, regarding volatile changes, a faster ion flux occurs in a high ion mobility and redox rate regime, although these changes do not persist over time.

Finally, **Figure S11c** demonstrates that optical changes in the structure reach a saturation point in the absence of counteracting pulses with opposite polarity. This indicates a finite number of achievable optical states within the structure, constrained by the minimum feasible separation between each state.

1.3. Thermal Characteristics of Memristor

One of the main conclusions of the conducted research was to demonstrate that, in memristors with well-defined optical resonance, optical readout offers significantly more robust and accurate insights into the device's resistance state compared to electrical measurements. From this perspective, thermal effects may potentially impact the device's response, as temperature variations can alter both the optical cavity's physical dimensions and intrinsic material properties. Especially, that the relatively large currents observed in our system might initially suggest some sample heating. However, this direct correlation is, in fact, misleading in this context. Given the size of the active area in our sample, the current density is approximately ~10⁻⁸ A/µm², which is substantially lower than the typical ~10⁻⁴ A/µm² reported in the literature for comparable ECM systems.^[41]^ Consequently, if macroscopic thermal effects are present, they are likely to be considerably weaker than in other reported systems. Additionally, thermal effects cannot account for the observed directional dependence of optical changes with respect to the polarity of the applied voltage (e.g., Figure 4b in the manuscript), and the dynamics of optical changes also occur more rapidly than would be expected from macroscopic thermal effects alone.

On the other hand, in the microscopic scale, the local temperature rise associated with the current flow might affect the optical properties, but in a different manner. As temperature increases, the mobility of ions, such as Ag or Cu, within the electrolyte also increases. This enhanced ion mobility leads to faster migration, allowing for more stable filament formation.^[27]^ Higher temperatures tend to suppress branched growth, favouring more compact, controlled filaments due to increased diffusion rates. Elevated temperatures can also enhance the redox rates, accelerating the deposition and dissolution processes that drive filament formation.

To verify the influence of the temperature on the proposed memristor structure, we conducted tests to examine how a global increase in the sample temperature would affect its optical response (**Figure S12**). In a straightforward experiment, we artificially increased the sample temperature by 30 °C, and observed that the resonance peak shifted toward shorter wavelengths, resulting in optical changes (Ψ, reflection coefficient) similar to those observed under positive voltage application. This suggests that the elevated temperature likely decreases the effective optical refractive index of the SiO_2_ switching layer, rather than expanding the width of the optical cavity. No memory effects were observed once the system returned to its initial temperature.

1.3. Additional Figures

| 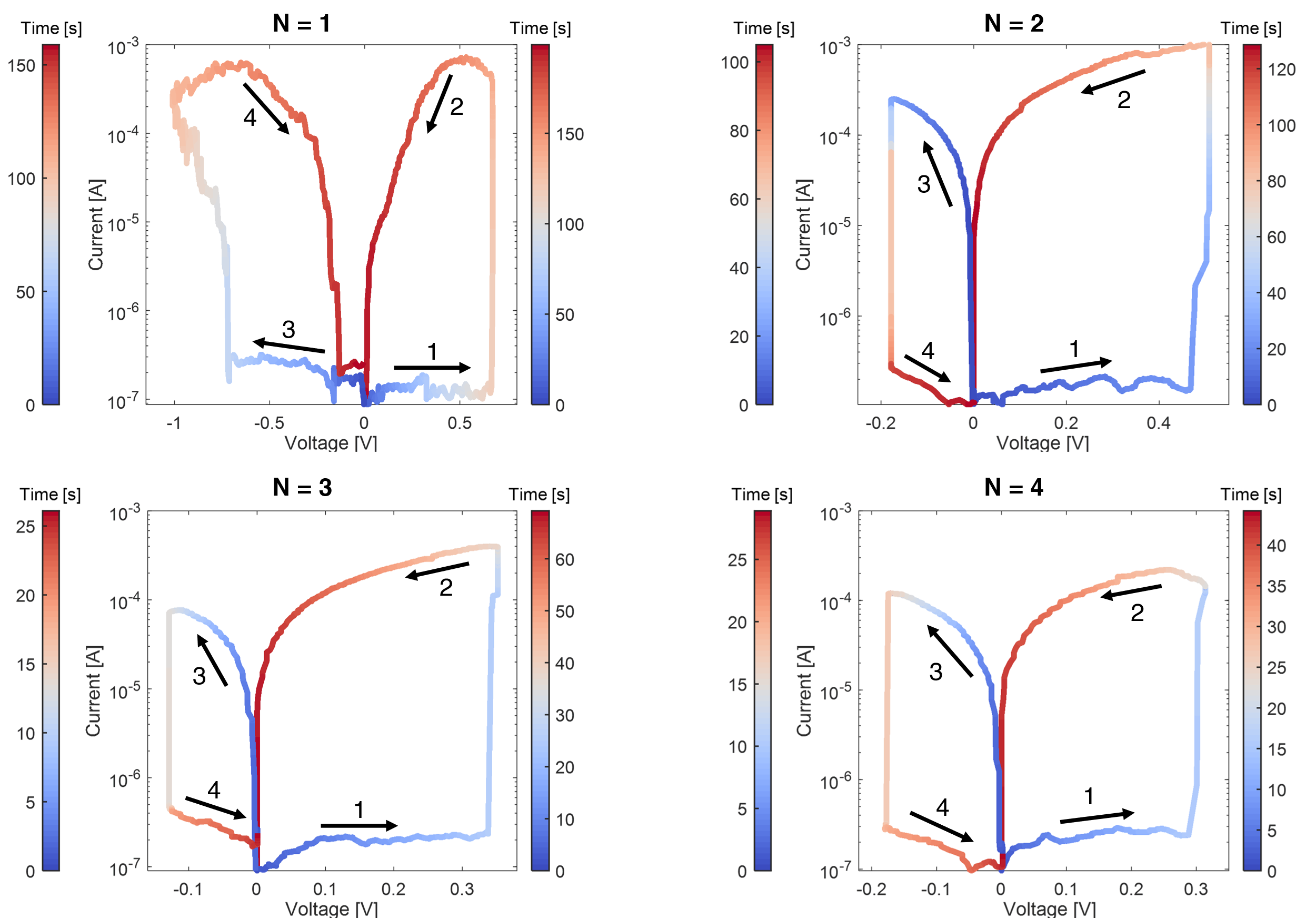 |
| --- |

**Figure S1. Memristive behavior of Ag/SiO_2_/Ag stack.** Current-voltage curves recorded for the first 4 cycles. Color transitions from blue to red signify the progression of time, and arrows show the direction of bias change. The left timescale corresponds to a negative voltage, while the right timescale to positive. In cycles 2, 3, and 4, the structure exhibits a hysteresis loop with a shape typical of a memristor.

| 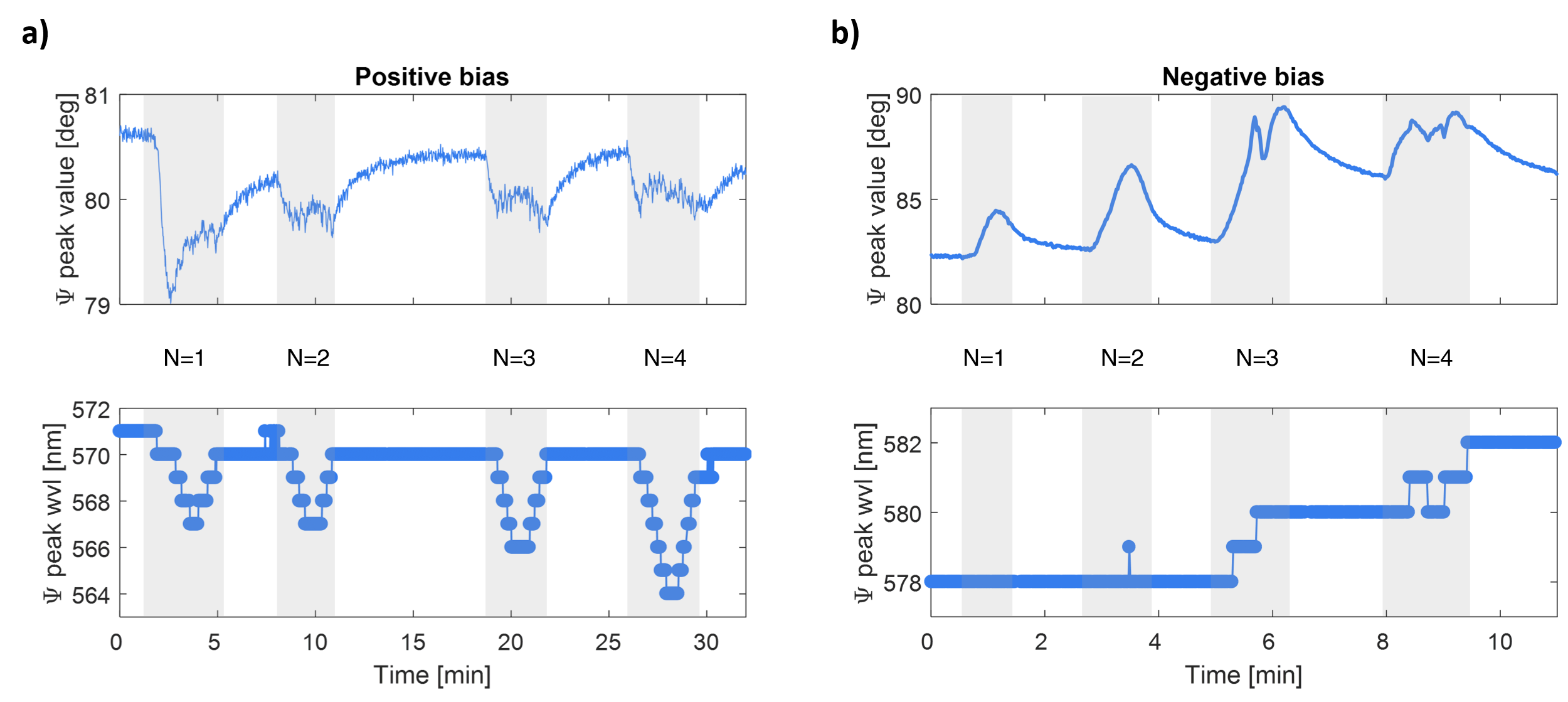 |
| --- |

**Figure S2. Temporal analysis of the Ψ peak characteristics.** The impact of the applied a) positive and b) negative voltage on peak value (top row) and peak spectral position (bottom row). The shaded areas represent the time interval at which the voltage was applied. Depending on the polarization voltage, two different characteristics are obtained.

| 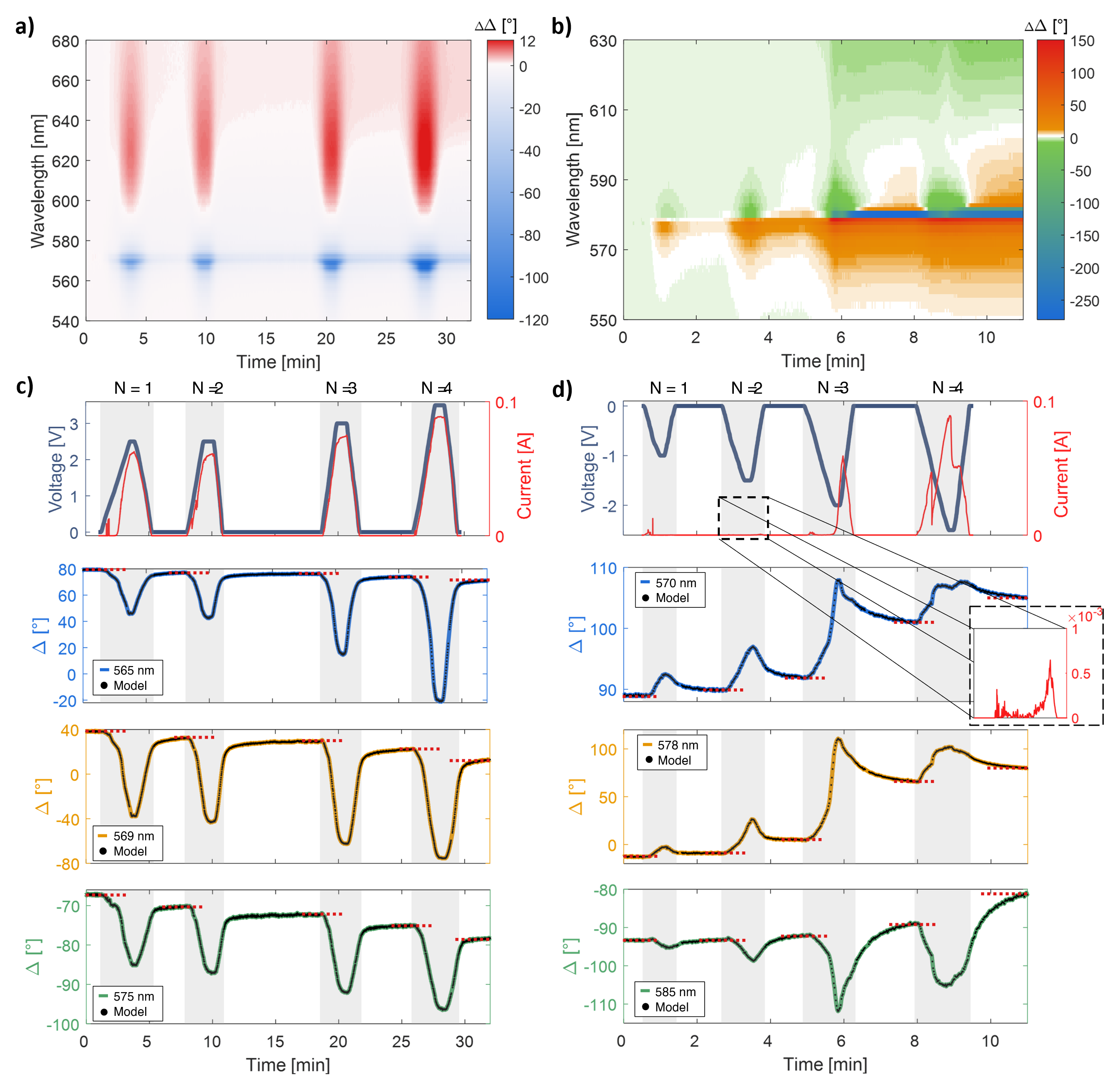 |
| --- |

**Figure S3. The device's electric and optical response.** The influence of the positive (a, c) and negative voltage (b, d) on the phase difference Δ. In a) and b), we show colormaps representing changes in Δ as a function of time and wavelength. c) and d) illustrate the time dependence of current and voltage, along with the corresponding Δ functions, measured for wavelengths of 565 nm, 569 nm, and 575 nm, respectively. The measured deltas are shown together with the developed model (black dotted line).

**
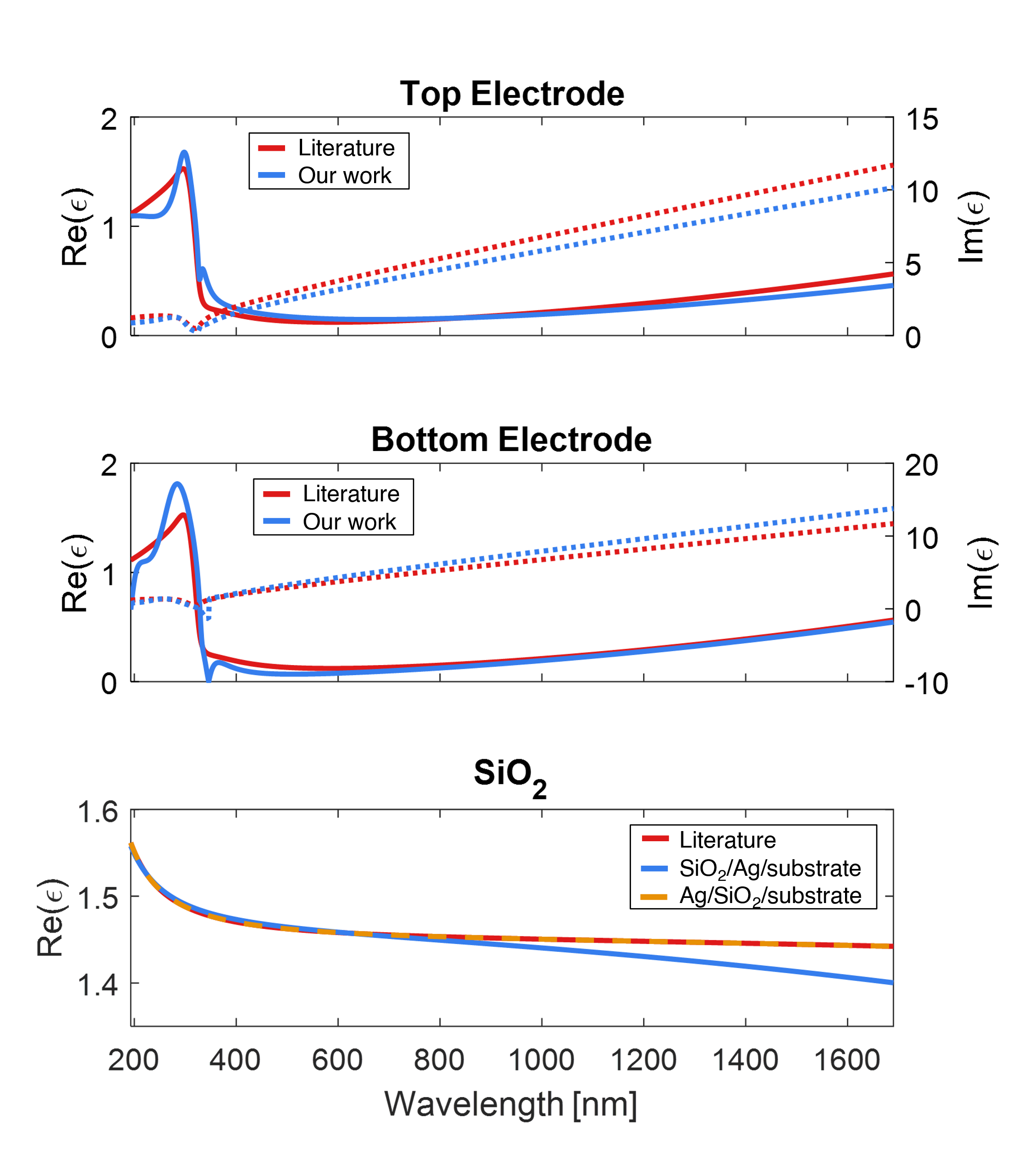
**

**Figure S4.** **Optical properties of freshly fabricated structure.** The refractive index (solid lines, left axis) and extinction coefficient (dotted lines, right axis) were extracted from the ellipsometric measurements. The reference data has been taken from [Palik, E. D. Handbook of Optical Constants of Solids; Academic Press: Orlando, FL, 1985.]. The optical constants of the SiO₂ layer (bottom row) vary depending on whether the layer was deposited directly on glass (orange, dotted line) or on a silver bottom electrode. We attribute the observed changes in the latter case to the presence of silver nanoclusters within the SiO₂ layer, resulting from the migration of Ag atoms during the evaporation process. Notably, the deposition of a top electrode did not have a similar effect on the optical properties of the SiO₂ layer.

| 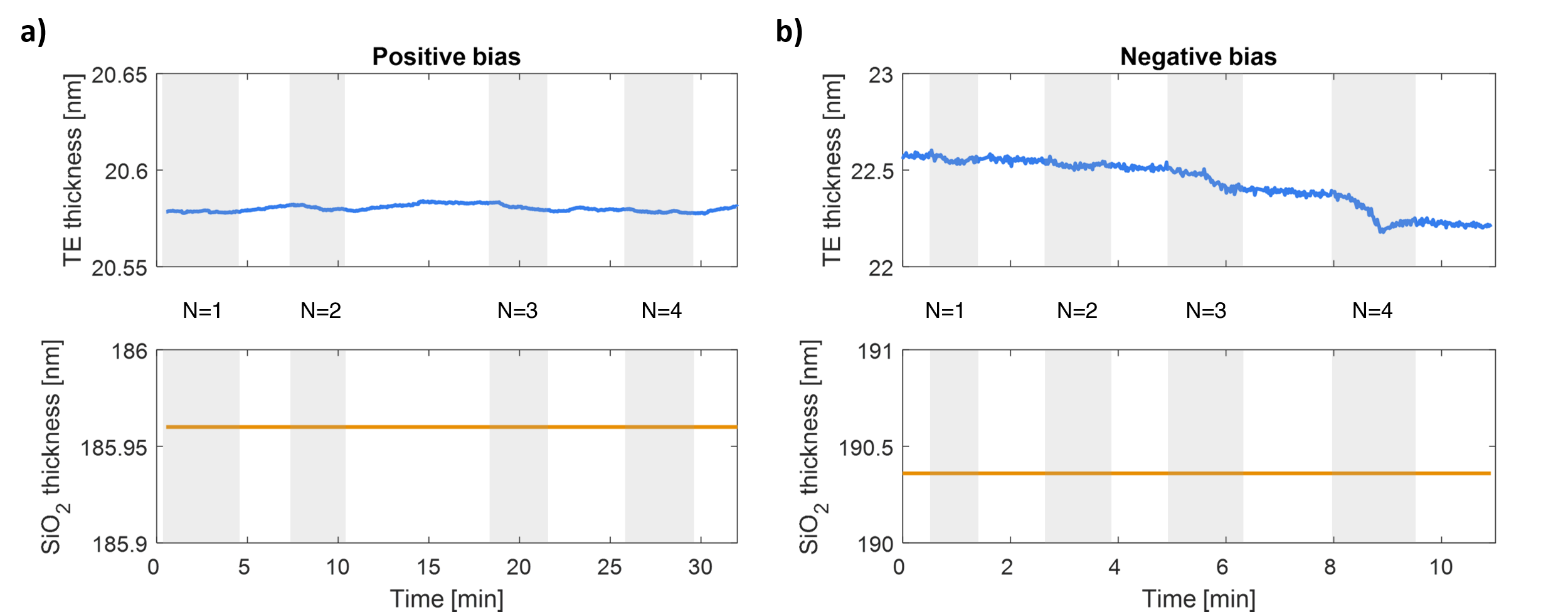 |
| --- |

**Figure S5. Physical properties of structure under electric modulation.** Time evolution of thickness of top electrode (top row) and SiO_2_ switching layer (bottom row) as a result of applied voltage. The data has been extracted from the ellipsometric model. Grey areas correspond to moments when the bias is varied. Judging from the shape of the SiO₂ thickness curves, the ellipsometric measurement or the created model is not sensitive enough to detect and indicate any notable changes in the switching layer. The SiO₂ thickness value obtained in the preliminary fitting procedure remained the most suitable throughout the entire in situ measurement.


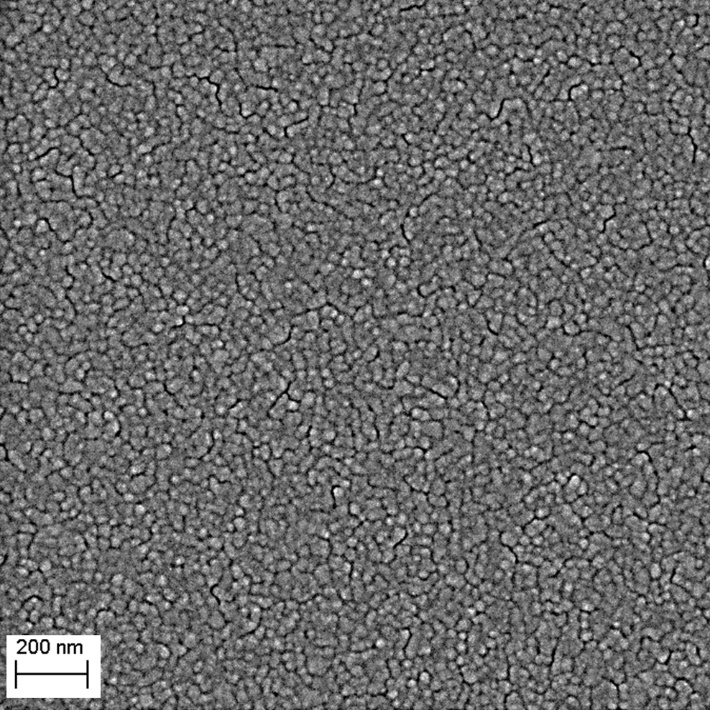


**Figure S6.** **Morphology of the top electrode.** SEM image of the silver electrode of 20 nm thickness. The scale bar corresponds to 200 nm. The surface of the layer exhibits visible percolation pathways and discontinuities.

**
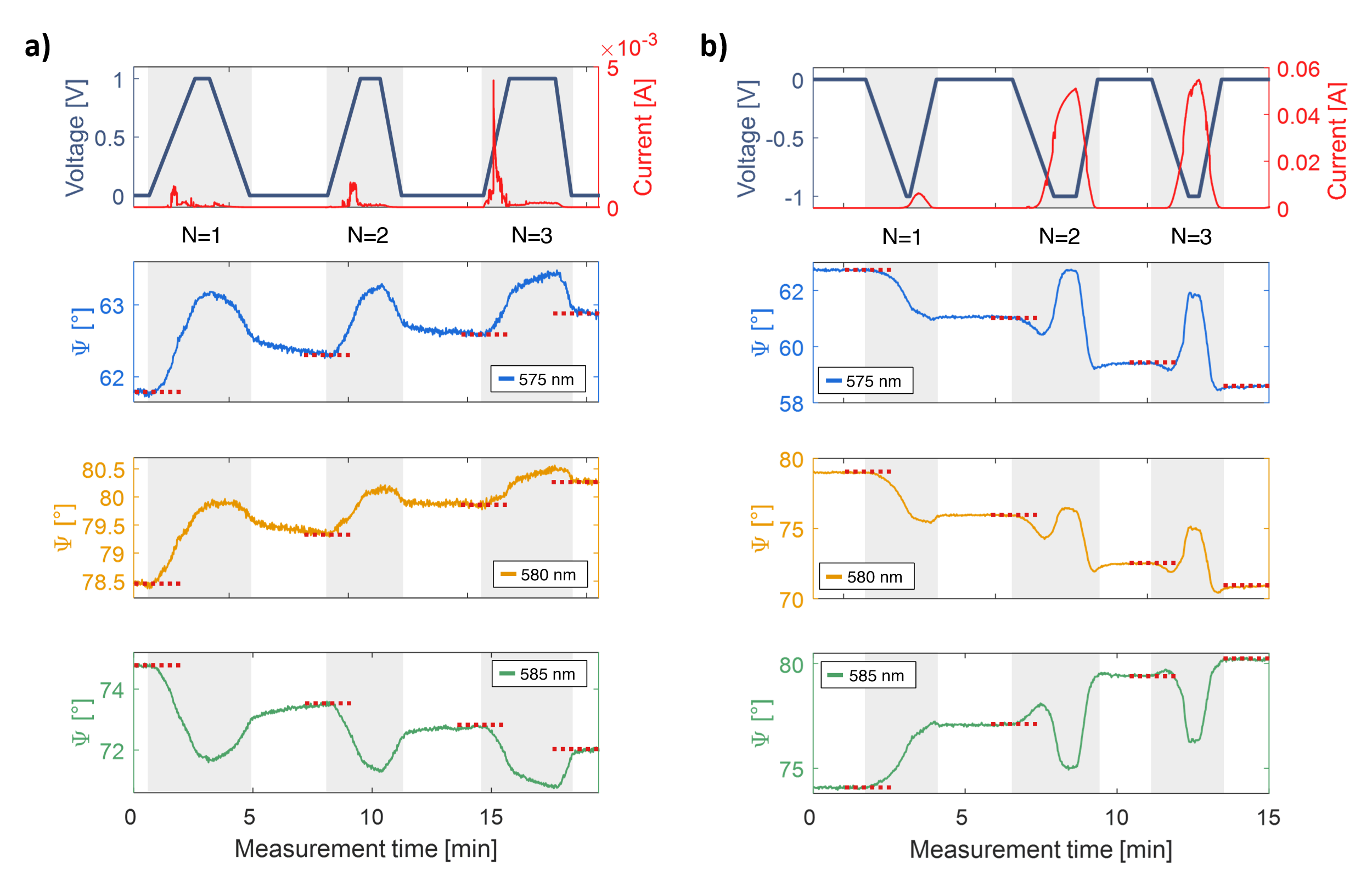
**

**Figure S7. The electric and optical response of Ag/SiO_2_/Au memristor system.** The influence of the positive (a) and negative voltage (b) on the flowing current (top row) and Ψ functions. The Ψ curves were collected under illumination at 65°, and for wavelengths of 575 nm, 580 nm, and 585 nm, respectively. Similar to the system with a silver bottom electrode, both volatile and nonvolatile changes were detected.


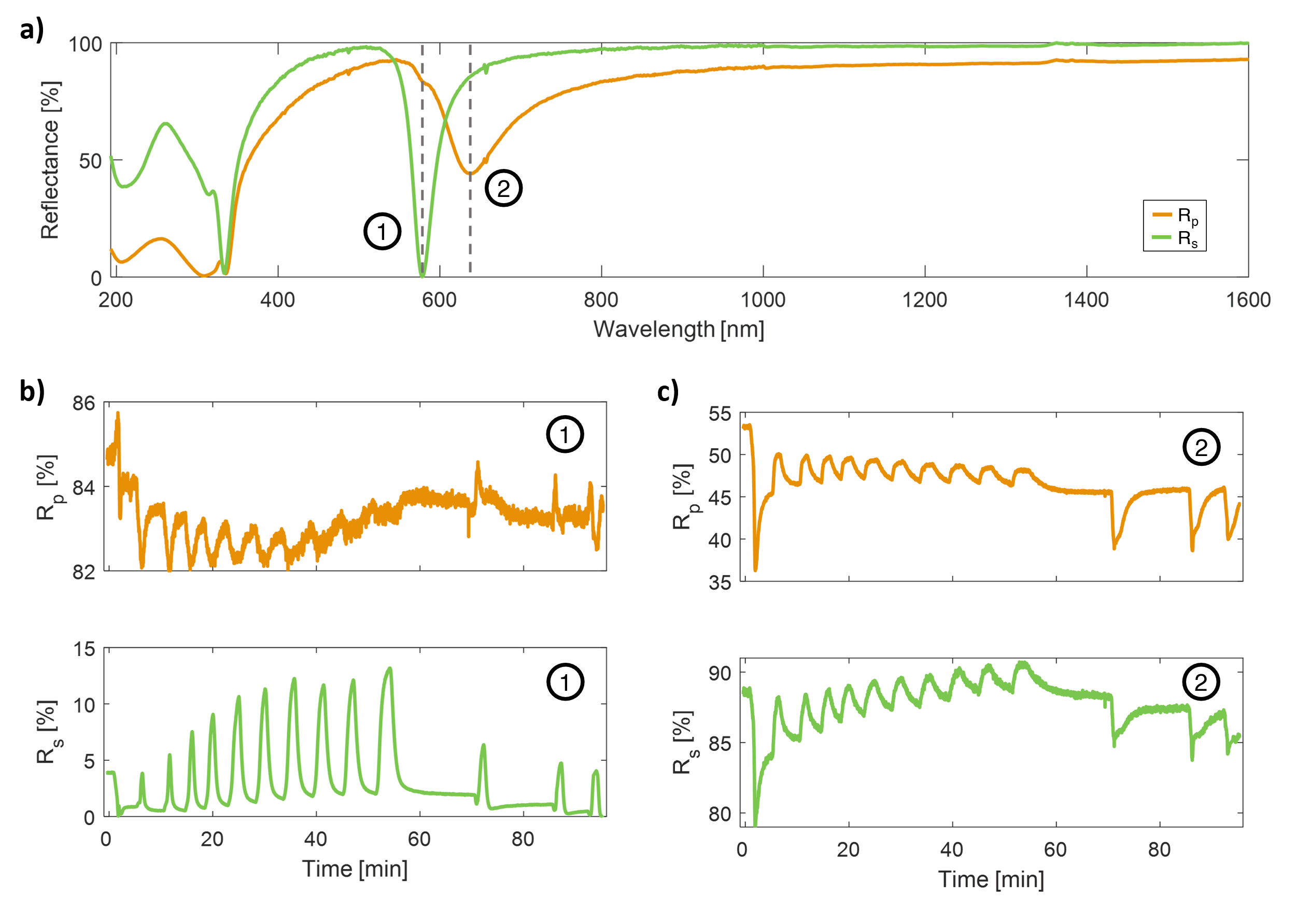


**Figure S8. Reflectance modulation under external bias.** a) The reflectance spectrum of the memristor structure investigated for different polarizations. The s- and p-polarisation resonance wavelengths are marked at 578 nm and 637 nm, respectively. b) Time trace of the reflectance for different polarizations at p-polarization wavelength resonance. c) Time trace of the reflectance for different polarizations at s-polarization wavelength resonance.


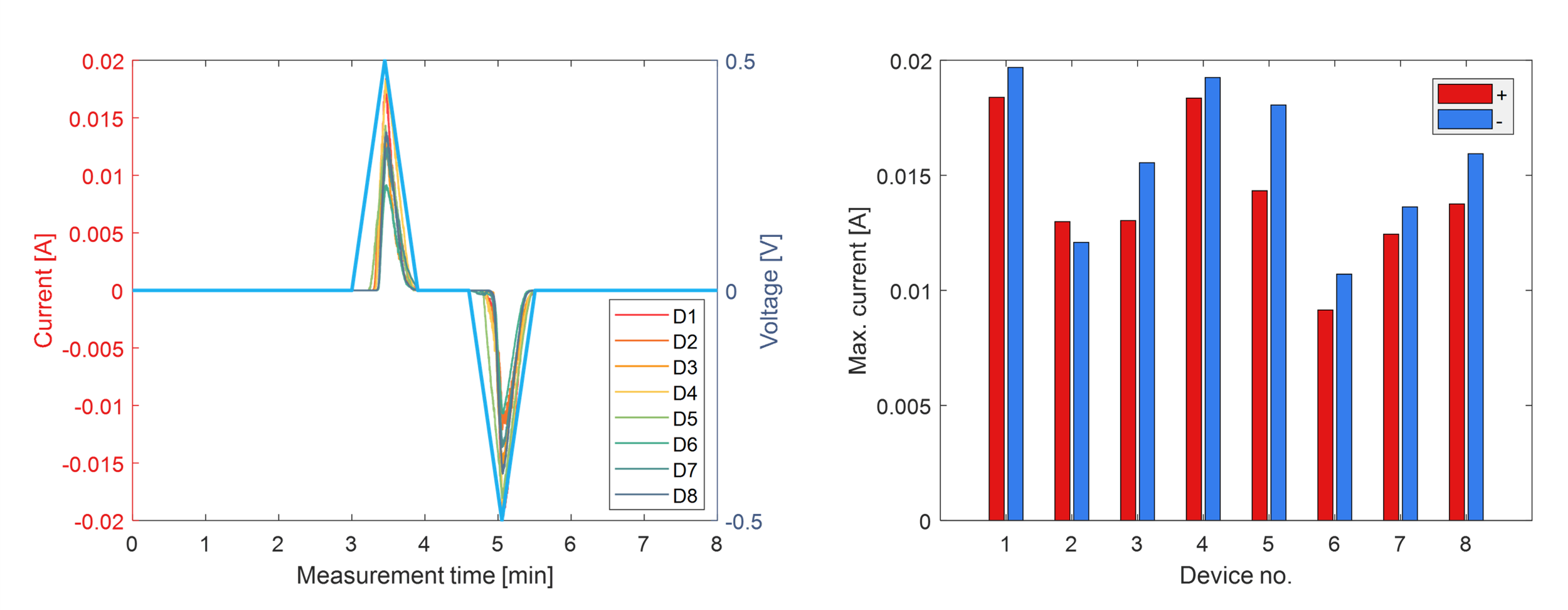


**Figure S9.** **Device-to-device variability in electrical performance.** a) Time-dependent I-V characteristics for eight newly fabricated devices. The blue line represents the applied voltage, while the colored lines denote current responses. b) Corresponding bar graph of maximum current values observed in each device as a function of voltage polarity. Notably, the current curves exhibit variability not only in peak values but also in the onset time of the current increase.


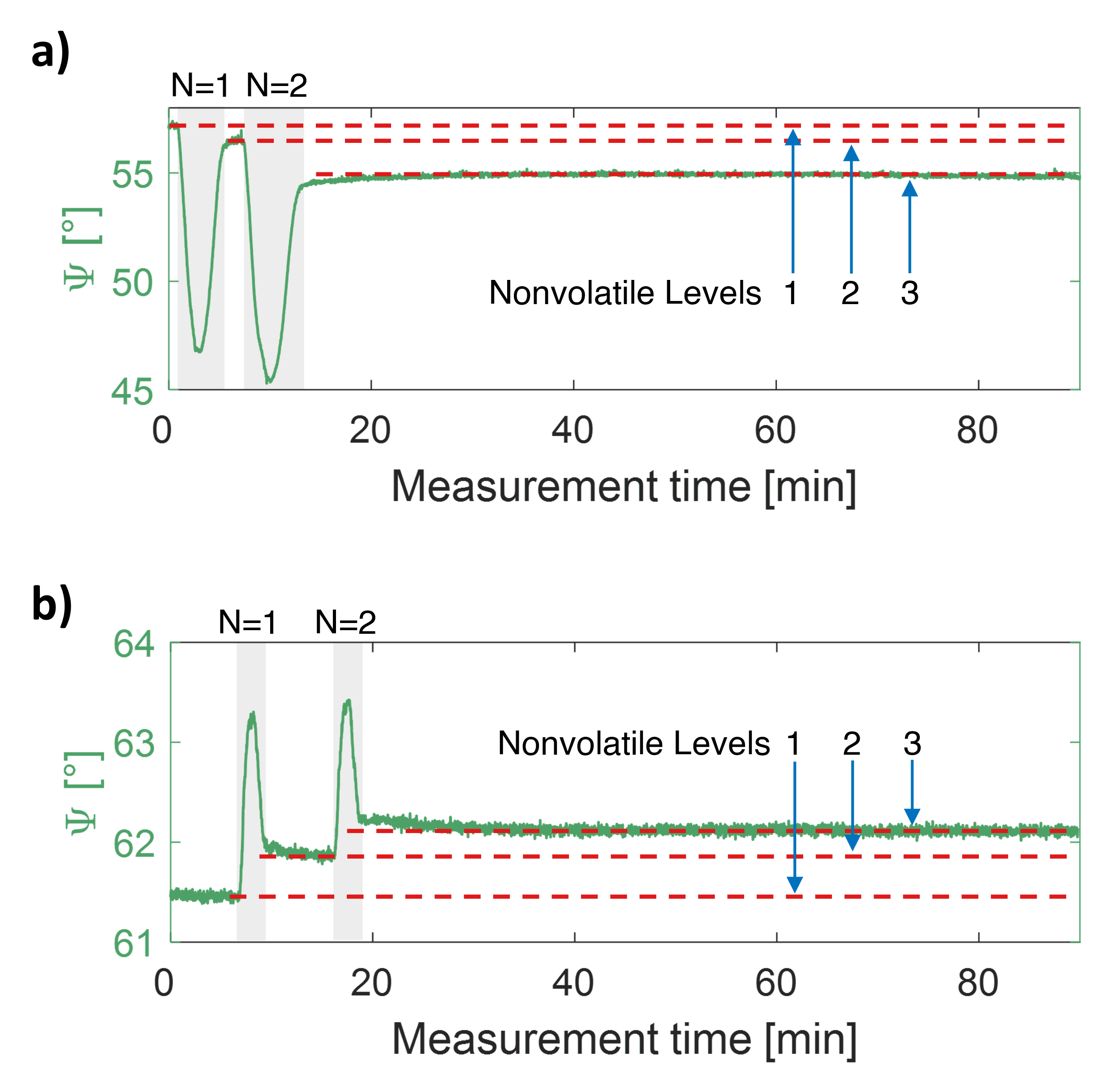


**Figure S10.** **Long-term stability of nonvolatile optical levels in memristor.** Time-dependent Ψ functions obtained under negative (a) and positive (b) voltage, recorded following stimulation with two electrical pulses. Time-dependent Ψ functions obtained under negative (a) and positive (b) voltage, recorded following stimulation with two electrical pulses. After voltage removal, the Ψ curves remained stable throughout a one-hour measurement period, with distinct optical levels staying well-separated and unchanged.


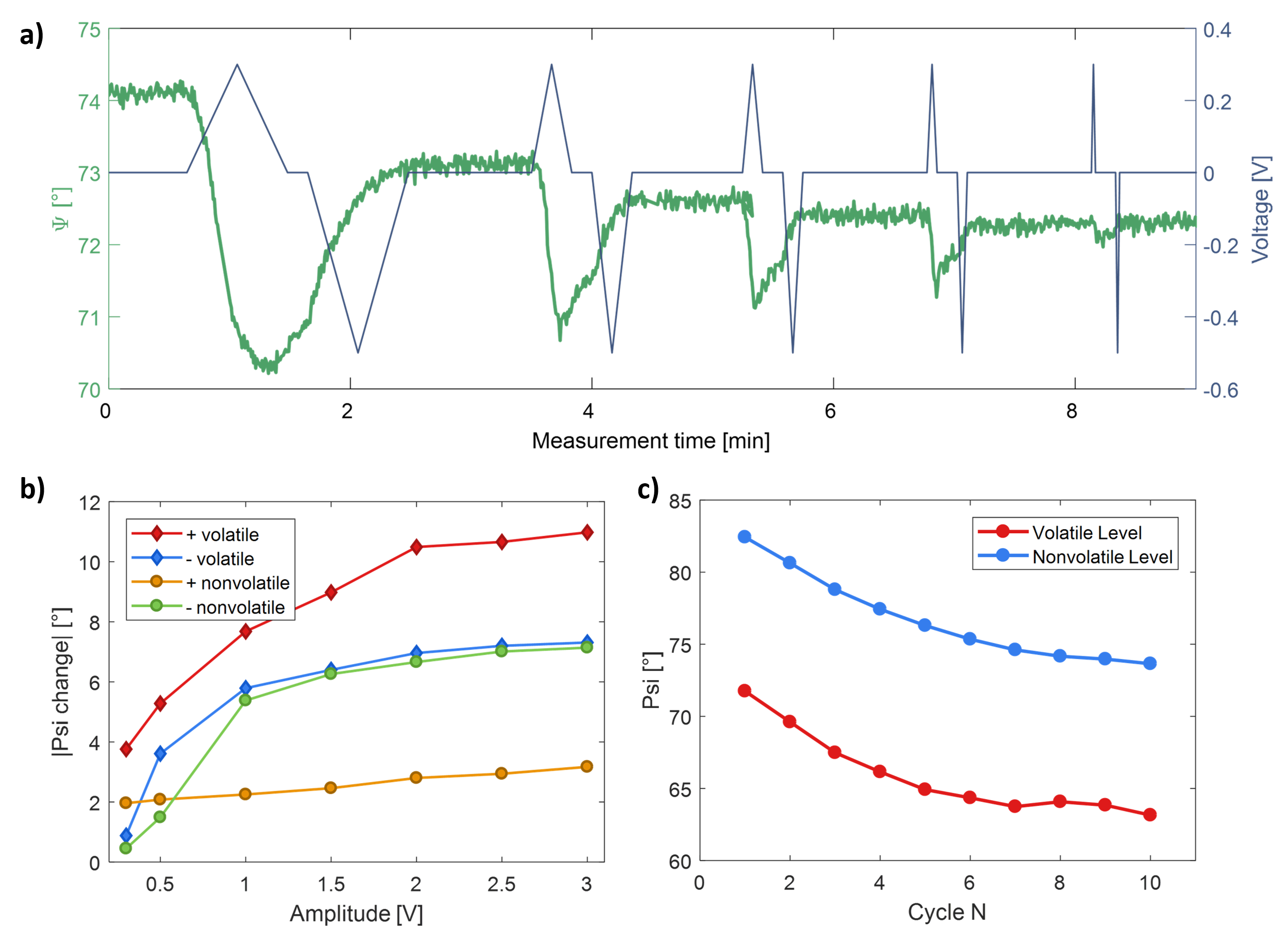


**Figure S11.** **Influence of pulse parameters on memristor optical response**. a) The time dependence of the Ψ function modulated by electrical pulses with durations of 50 s, 20 s, 10 s, 5 s, and 2 s, and amplitudes of +0.3 V and -0.5 V, respectively. b) The averaged values of volatile and nonvolatile changes in the Ψ function, calculated over different pulse lengths, as a function of the amplitude of the applied electrical pulses. c) The progression of volatile and nonvolatile Ψ levels in response to sequential electrical pulses of 2 V.


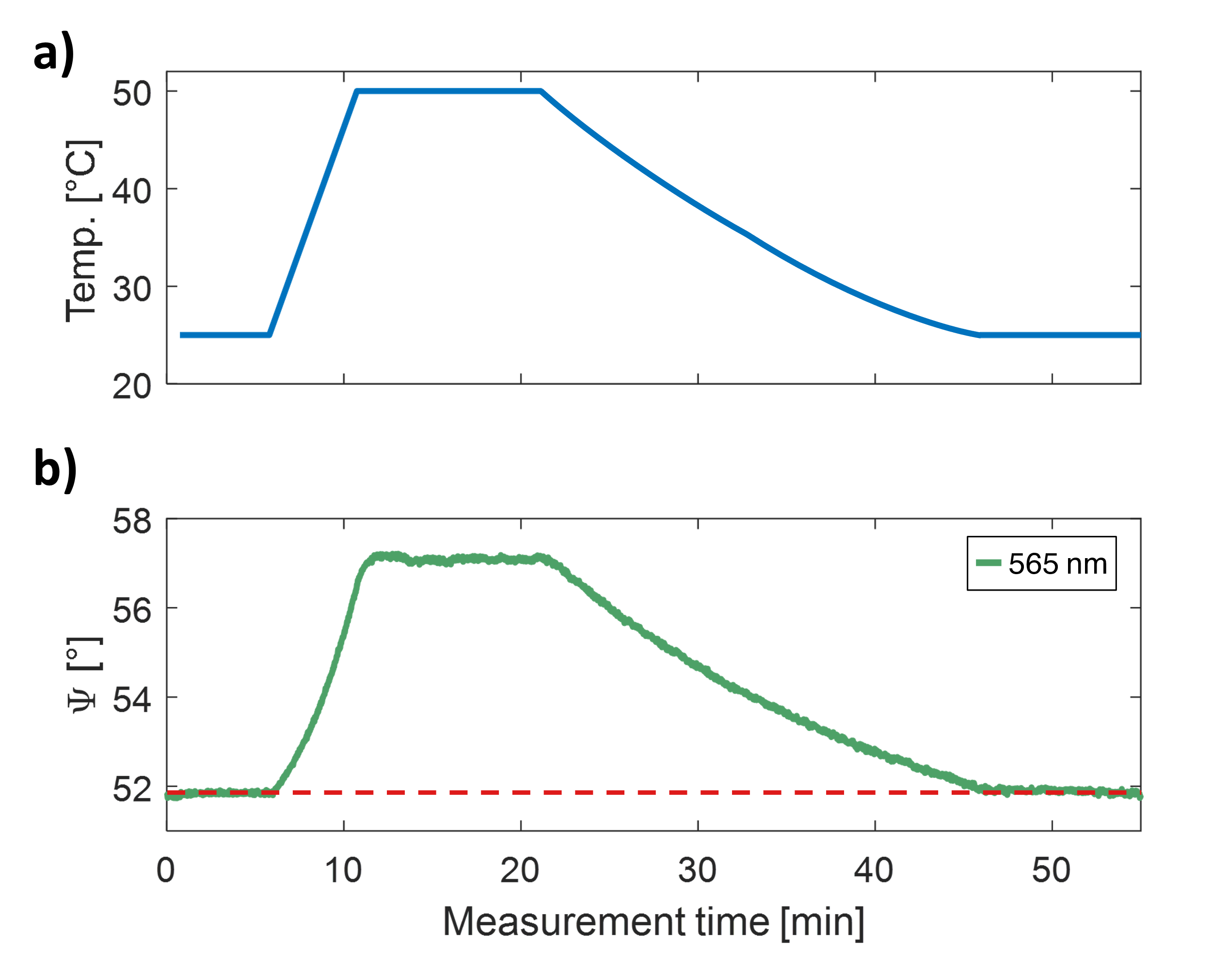


**Figure S12.** Thermal Characteristics of the Memristor. a) Temperature profile of the stabilized sample stage. b) Corresponding measured changes in the Ψ function.
